# Vaccine-Induced Immunity Elicited by Microneedle Delivery of Influenza Ectodomain Matrix Protein 2 Virus-like Particle (M2e VLP)-Loaded PLGA Nanoparticles

**DOI:** 10.3390/ijms241310612

**Published:** 2023-06-25

**Authors:** Keegan Braz Gomes, Sharon Vijayanand, Priyal Bagwe, Ipshita Menon, Akanksha Kale, Smital Patil, Sang-Moo Kang, Mohammad N. Uddin, Martin J. D’Souza

**Affiliations:** 1 Center for Drug Delivery and Research, Vaccine Nanotechnology Laboratory, College of Pharmacy, Mercer University, Atlanta, GA 30341, USA; 2 Center for Inflammation, Immunity, and Infection, Institute for Biomedical Sciences, Georgia State University, Atlanta, GA 30303, USA

**Keywords:** influenza, vaccine, microneedles, nanoparticles, VLP, PLGA

## Abstract

This study focused on developing an influenza vaccine delivered in polymeric nanoparticles (NPs) using dissolving microneedles. We first formulated an influenza extracellular matrix protein 2 virus-like particle (M2e VLP)-loaded with poly(lactic-co-glycolic) acid (PLGA) nanoparticles, yielding M2e5x VLP PLGA NPs. The vaccine particles were characterized for their physical properties and in vitro immunogenicity. Next, the M2e5x VLP PLGA NPs, along with the adjuvant Alhydrogel^®^ and monophosphoryl lipid A^®^ (MPL-A^®^) PLGA NPs, were loaded into fast-dissolving microneedles. The vaccine microneedle patches were then evaluated in vivo in a murine model. The results from this study demonstrated that the vaccine nanoparticles effectively stimulated antigen-presenting cells in vitro resulting in enhanced autophagy, nitric oxide, and antigen presentation. In mice, the vaccine elicited M2e-specific antibodies in both serum and lung supernatants (post-challenge) and induced significant expression of CD4^+^ and CD8^+^ populations in the lymph nodes and spleens of immunized mice. Hence, this study demonstrated that polymeric particulates for antigen and adjuvant encapsulation, delivered using fast-dissolving microneedles, significantly enhanced the immunogenicity of a conserved influenza antigen.

## 1. Introduction

Each year, there are approximately three to five million severe cases of influenza worldwide, causing between 290,000 and 650,000 deaths [1]. The influenza virus belongs to the Orthomyxoviridae family and can be classified into four types: A, B, C, and D, with influenza A viruses (IAVs) being the primary cause of previous pandemics or seasonal epidemics [1,2]. The negative strain RNA virus contains eight genes that code for 11 proteins, including hemagglutinin (HA), neuraminidase (NA), matrix protein 2 (M2), and nucleoprotein (NP) [3]. HA allows the virus to bind to the host cell and aids in viral entry. NA assists in cleaving progeny virions on the infected cells, M1 aids in the budding of the virus from the plasma membrane of infected cells, and M2 is a proton ion channel that facilitates the maintenance of pH during viral entry and viral replication in the host cell [2,3].

Vaccination remains the most effective strategy against influenza [4]. Since 2014, quadrivalent vaccines containing one H1N1, one H3N2, and two influenza B strains (inactivated or live-attenuated) have been available to the public [5]. These vaccines elicit neutralizing antibodies against strain-specific epitopes on the globular head of the HA surface glycoprotein [6]. However, HA is highly susceptible to antigenic drift resulting in the accumulation of mutations, which can make these HA antigenically different from HA in previously circulating strains [7]. As a result, seasonal vaccine efficacy can vary widely by year, depending on the antigenic similarities between the vaccine strains and the circulating strains at the time [6]. Moreover, repeated vaccination has been shown to reduce vaccine efficacy, resulting in lower protection after consecutive vaccinations [8,9]. Therefore, there has been a push toward developing a universal pan-influenza A vaccine that can confer cross-protectivity against all IAVs [6]. 

The extracellular domain of the matrix protein 2 (M2e) of the influenza virus has gained much interest as a target for the development of a universal influenza A vaccine [10,11,12,13]. M2 is a 97-amino acid ion channel located on the membrane of the influenza virus and plays a significant role during viral entry by maintaining pH [14]. The extracellular domain of M2, M2e, is a highly conserved 23-amino acid sequence that extends into the extracellular domain of the viral membrane [10,15]. However, M2e is shielded by larger and highly abundant HA and NA glycoproteins on the influenza viral surface, which reduces its interactions with immune cells upon infection [10]. Therefore, to increase the immunogenicity of M2e, M2e has been previously conjugated to various immunogenic carriers such as hepatitis B core particles, human papillomavirus L proteins, and liposomes [16]. Alternatively, M2e has also been genetically engineered into virus-like particles (VLPs) containing tandem repeats of M2e sequences originating from various human, avian, and swine influenza A viruses, deemed M2e5x [17]. The main advantage of a VLP construct is that it mimics the structure and organization of the pathogen in its native state but lacks any genetic material and is therefore considered a safe candidate for vaccine development [18]. Currently, there are several approved VLP vaccines on the market, including Engerix^®^ and Sci-B-Vac^TM^ (hepatitis B virus), Gardasil^®^ (human papillomavirus), and Mosquirix^TM^ (malaria) [18]. Therefore, developing an influenza vaccine that utilizes tandem repeats of the M2e in a VLP construct with an increased M2e epitope density can potentially induce stronger M2e-specific immunity [10].

To further improve the immunogenicity of an antigen, particulate delivery systems such as liposomes and polymeric microparticles can be used [19]. Particulate systems are advantageous as they can mimic the size of the pathogens, allowing for improved immune recognition and internalization by antigen-presenting cells (APCs) [19,20,21]. Additionally, a sustained-release particulate delivery system can also increase antigen retention in lymphoid tissues, allowing for continuous antigen presentation in local lymph nodes (LNs) for extended periods [19,22]. Poly(lactic-co-glycolic acid) (PLGA) is one of the most widely used synthetic polymers for drug delivery due to its sustained release properties and biocompatible nature [23]. Additionally, the safety of PLGA has been recognized by the United States Food and Drug Administration (FDA) and the European Medicines Agency and is currently included by the FDA as a pharmaceutical excipient [23].

Previous studies have shown that microparticles (polymeric, liposomes, etc.), themselves, can confer adjuvant capabilities on their own [19,24]. Delivery systems utilizing nanoparticles (NPs) or microparticles (MPs) can also be modified or combined with immunopotentiators, such as adjuvants, to further help boost intrinsic immunity by stimulating APCs against pathogens such as influenza [19,25]. Alhydrogel^®^, commonly referred to as alum, is one of the most widely used licensed adjuvants, specifically against influenza [26,27]. It produces a depository effect at the administration site, which recruits immune cells and enhances antigen presentation [27,28]. Monophosphoryl lipid A^®^ or MPL-A^®^ is an emulsion-based low-toxicity derivative of lipopolysaccharide (LPS) [29]. MPL-A^®^ is a toll-like receptor 4 (TLR-4) agonist and has been shown to skew the immune response towards Th1, which induces cell-mediated immunity [30]. It has also been shown to stimulate immunoglobulin A (IgA) antibody responses and has been used in past influenza vaccines [31].

Lastly, the immunization route plays a significant role in vaccine-induced immunity. The most common route for vaccines is the intramuscular route. However, recent vaccine research has focused on alternate routes using non-injectable strategies. One of these immunization routes targets the skin, which is the first line of defense against pathogens [32]. The epidermis and dermis of the skin contain Langerhans cells (LCs) and dermal dendritic cells, which are antigen-presenting cells (APCs) capable of activating T and B lymphocyte pathways [25,33,34]. However, as proteins are large in size and hydrophilic, they cannot passively diffuse across the skin. To overcome this limitation, several techniques can be used to facilitate the transdermal delivery of proteins, including dissolving microneedles. Dissolving microneedles consist of water-soluble or biodegradable polymers encapsulating a drug within the matrix [13,32]. Once the patch is inserted into the skin, the needles completely dissolve, releasing the encapsulated payload [32,34,35]. Vaccination through microneedle technology is an attractive approach due to the active targeting of immune cells in the skin [25,33,34,35]. Transdermal vaccines have been previously developed and tested in animals to protect against diphtheria toxoid, anthrax, hepatitis B, and influenza [25,32]. Our group has extensively explored the use of alternate routes for vaccine delivery as well as various polymeric particulate systems to deliver various antigens for targeting pathogens such as influenza, respiratory syncytial virus (RSV), SARS-CoV-2, Zika, human papillomavirus (HPV), and gonorrhea [13,25,33,34,36,37,38,39,40].

In this study, we found that M2e5x VLP encapsulated in PLGA nanoparticles induced a strong in vitro immunogenicity in antigen-presenting cells (APCs) through induction of autophagy and nitric oxide, and heightened antigen presentation. In vivo, the vaccine nanoparticles administered to mice using dissolving microneedles in the skin elicited M2e-specific antibody and cell-mediated responses. Hence, our proof-of-concept study demonstrated our influenza vaccine to be a suitable candidate for a potential universal pan-influenza A vaccine.

## 2. Results

### 2.1. Physical Characterization of Vaccine Nanoparticles

**Figure 1:**
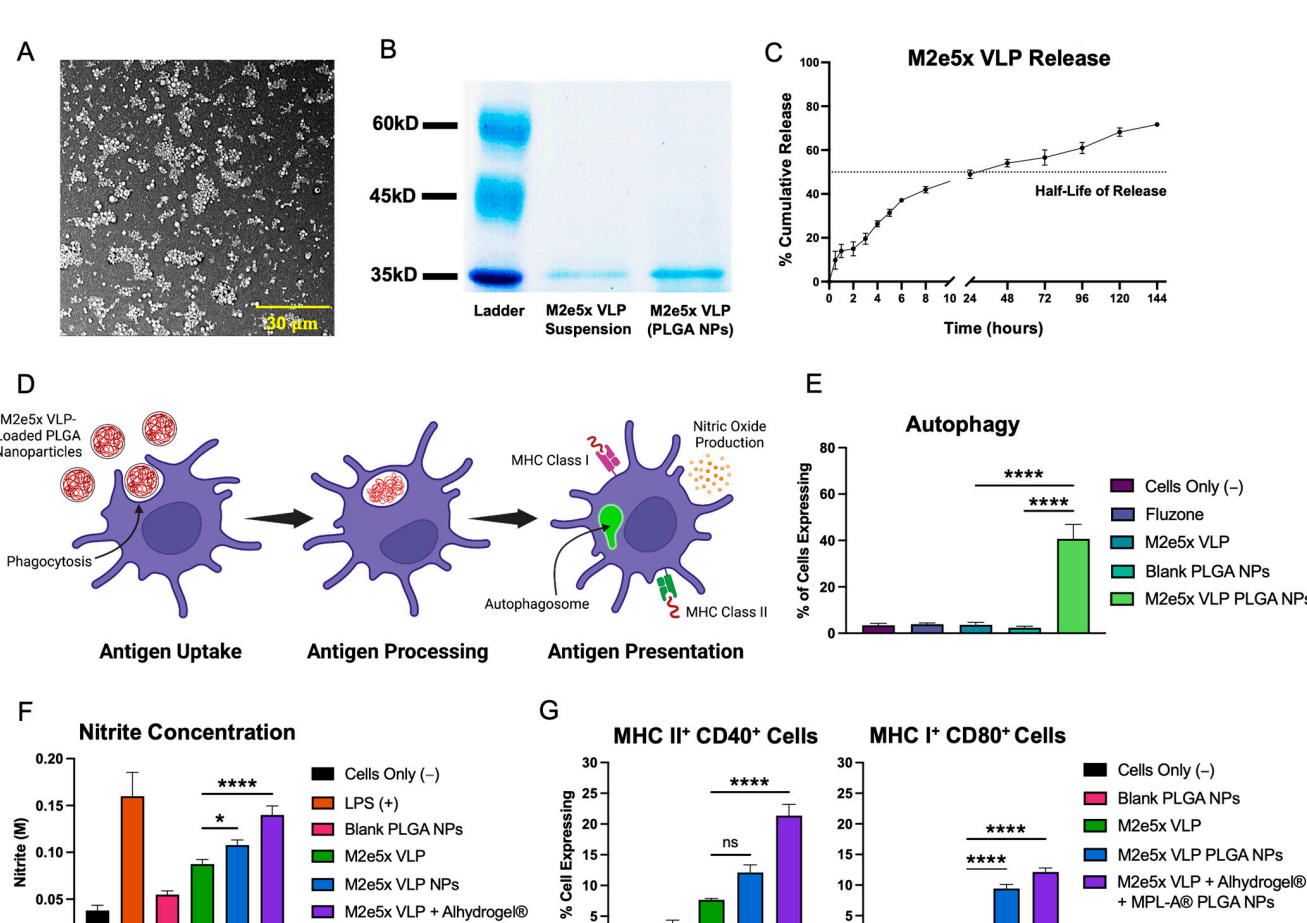
Formulation characterization and in vitro immunogenicity of M2e5x VLP PLGA NP vaccine. (**A**) Scanning electron microscopy (SEM) image of M2e5x VLP-loaded PLGA nanoparticles. (**B**) Gel of M2e5x VLP suspension compared to M2e5x VLP extracted from PLGA NPs confirms that the formulation of M2e5x VLP PLGA NPs does not affect the integrity of the VLP. (**C**) Release of M2e5x VLP from PLGA NPs over the course of 144 h (6 days). There was an initial burst release observed, followed by a sustained release profile. The half-life of release in PBS is approximately 24 h. (**D**) Schematic showing antigen uptake, processing, and presentation in a single dendritic cell (created with BioRender.com). The particulate antigen is first taken up by phagocytosis by DCs and further processed resulting in the heightened production of autophagosomes and nitric oxide, and surface presentation of the antigen on MHC class I and II molecules. (**E**) Autophagy in cells stimulated by various treatments. Comparisons are made between M2e5x VLP PLGA NPs, and M2e5x VLP and Blank PLGA NPs. (**F**) Nitrite concentrations quantifying nitric oxide (reduced to nitrite) produced by DCs pulsed with various treatments. (**G**) Co-expression of complimentary MHC II/CD40 and MHC I/CD80 APC surface markers using flow cytometry. Comparisons are made between M2e5x VLP, and M2e5x VLP PLGA NPs and M2e5x VLP + Alhydrogel^®^ + MPL-A^®^ PLGA NPs unless specified otherwise. All data are presented as the mean ± SD. * *p* < 0.05, **** *p* < 0.0001, and “ns” indicates not significant.

The M2e5x VLP-loaded PLGA nanoparticles product yield was 91.7%, with an average particle size of 580.2 ± 20.2 nm, and zeta potential of −7.1 ± 0.44 mV. For Alhydrogel^®^-loaded PLGA nanoparticles, the yield was 92.6% with an average particle size of 457.8 ± 1.76 nm, and a zeta potential of 12 ± 0.25 mV. Lastly, the product yield of MPL-A^®^-loaded PLGA nanoparticles was 92.8%, with an average size of 339 ± 5.3 nm, and a zeta potential of 0.338 ± 0.16 mV. Scanning electron microscopy images of the M2e5x VLP PLGA NPs are shown in Figure 1A. All PLGA NPs were spherical in their morphology. The encapsulation efficiency of the M2e5x VLP PLGA NPs was found to be approximately 88%. Confirmation of antigen integrity was carried out using SDS PAGE (Figure 1B). The results of the gel showed identical bands near 37 kDa for the M2e5x VLP in suspension and M2e5x VLP extracted from PLGA NPs. Next, release data showed that approximately 50% of the M2e5x VLP was released from the PLGA particles in approximately 24 h (Figure 1C). After the initial burst release, the antigen continued to release in a sustained profile, with more than 75% cumulative release taking place by 144 h (6 days). The vaccine PLGA nanoparticle-loaded microneedles in the 10 × 10 array were found to be approximately 380 μm in length.

### 2.2. Vaccine Nanoparticles Elicit In Vitro Immunogenicity in Antigen-Presenting Cells

A schematic for antigen uptake, processing, and presentation is shown in Figure 1D. For induction of autophagy, M2e5x VLP PLGA NPs produced significantly higher amounts of autophagy compared to M2e5x VLP and Blank PLGA NPs (Figure 1E). M2e5x VLP + Alhydrogel^®^ + MPL-A^®^ PLGA NPs also showed significantly higher levels of nitrite production compared to Fluzone (marketed flu vaccine) and M2e5x VLP suspension (Figure 1F). M2e5x VLP + Alhydrogel^®^ + MPL-A^®^ PLGA NPs also showed a higher nitrite production compared to Blank PLGA NPs and M2e5x VLP PLGA NPs. For antigen presentation in DCs, M2e5x VLP + Alhydrogel^®^ + MPL-A^®^ PLGA NPs showed significantly higher co-expression of MHC II/CD40 compared to M2e5x VLP (Figure 1G). For the co-expression of MHC I/CD80 in DCs, M2e5x VLP PLGA NPs and M2e5x VLP + Alhydrogel^®^ + MPL-A^®^ PLGA NPs showed significantly higher expression compared to M2e5x VLP.

### 2.3. Vaccine Nanoparticles Administered Using Dissolving Microneedles Induce M2e-Specific Antibody Responses in Mice

**Figure 2:**
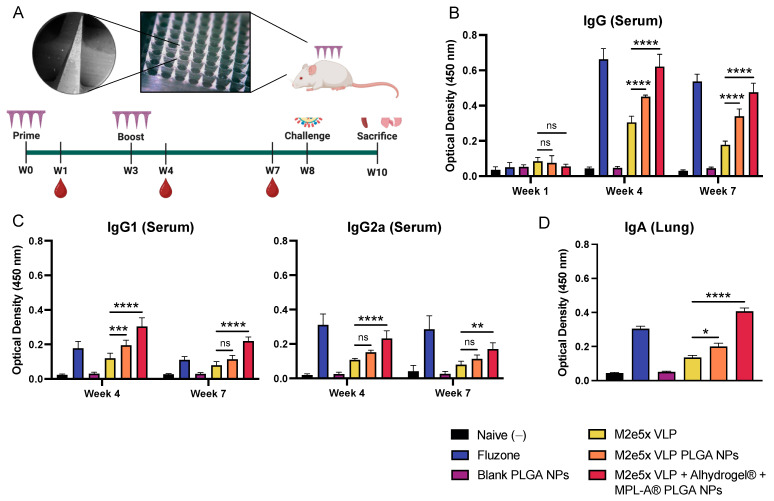
Antibody response induced in mice immunized by M2e5x VLP PLGA NPs using dissolving MNs. (**A**) Images of M2e5x VLP-loaded PLGA NPs incorporated into fast-dissolving microneedles. Study timeline with immunizations in weeks 0 and 3, blood collection in weeks 1, 4, and 7, sublethal challenge in week 8, and sacrifice and organ harvesting in week 10. (**B**) M2e-specific binding of serum from mice immunized with M2e5x VLP in suspension, in PLGA nanoparticles, or in PLGA nanoparticles with Alhydrogel^®^ + MPL-A^®^ PLGA NPs. Fluzone-coated wells were used to assess the binding of serum from Fluzone-immunized mice. (**C**) M2e-specific IgG1 and IgG2a serum binding. (**D**) M2e-specific IgA binding in the serum of immunized mice. Data are presented as the mean ± SD. Comparisons are made between M2e5x VLP and M2e5x VLP NPs and M2e5x VLP + Alhydrogel^®^ + MPL-A^®^ PLGA NPs. * *p* < 0.05, ** *p* < 0.01, *** *p* < 0.001, **** *p* < 0.0001, and “ns” indicates not significant.

Vaccine nanoparticle-loaded microneedle images (SEM and digital) and the study timeline are shown in Figure 2A. Mouse serums from weeks 1, 4, and 7 were analyzed to determine M2e-specific binding after a prime-boost immunization regimen (weeks 0 and 3). M2e-specific serum IgG is shown in Figure 2A. After one-week post-prime immunization, there was no difference in M2e binding of M2e5x VLP PLGA NPs and M2e5x VLP + Alhydrogel^®^ + MPL-A^®^ PLGA NPs compared to M2e5x VLP. In week 4, M2e5x VLP PLGA NP and M2e5x VLP + Alhydrogel^®^ + MPL-A^®^ PLGA NP groups showed significantly higher M2e-specific IgG binding compared to M2e5x VLP. Similarly, in week 7, M2e5x VLP PLGA NP and M2e5x VLP + Alhydrogel^®^ + MPL-A^®^ PLGA NPs showed higher IgG compared to M2e5x VLP. For IgG subtypes (Figure 2C), M2e5x VLP PLGA NPs showed higher IgG1 binding in week 4 compared to M2e5x VLP whereas M2e5x VLP + Alhydrogel^®^ + MPL-A^®^ PLGA NPs showed higher M2e-specific IgG1 binding in weeks 4 and 7. For IgG2a, there was no significantly higher M2e-specific binding observed between M2e5x VLP PLGA NPs and M2e5x VLP in weeks 4 and 7. In contrast, M2e5x VLP + Alhydrogel^®^ + MPL-A^®^ PLGA NP-immunized mice showed significantly higher IgG2a than M2e5x VLP. After sacrifice, lung tissues were homogenized to determine the levels of IgA in the lung supernatants. Both M2e5x VLP PLGA NPs and M2e5x VLP + Alhydrogel^®^ + MPL-A^®^ PLGA NPs showed significantly higher M2e-specific IgA than M2e5x VLP (Figure 2D). 

### 2.4. Vaccine Nanoparticles Administered in Mice Using Dissolving Microneedles Induce T Cell Responses in Mice

**Figure 3:**
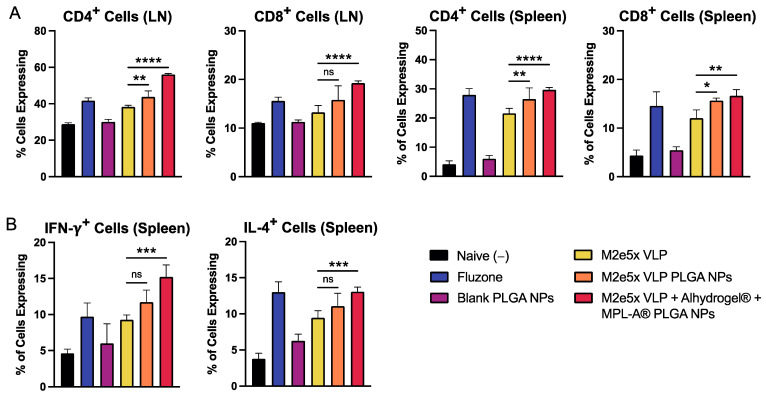
Antibody responses induced in mice immunized by M2e5x VLP PLGA NPs delivered using dissolving microneedles. (**A**) Expression of CD4^+^ and CD8^+^ cells in lymph node (LN) and spleens of immunized mice. M2e5x VLP antigen-immunized groups were pre-stimulated with M2e. Organs from mice immunized with Fluzone were pre-stimulated with quadrivalent Fluzone. (**B**) Expression of intracellular cytokines IFN-γ and IL-4 using flow cytometry. Data are presented as the mean ± SD. Comparisons are made between M2e5x VLP, and M2e5x VLP NPs and M2e5x VLP + Alhydrogel^®^ + MPL-A^®^ PLGA NPs. * *p* < 0.05, ** *p* < 0.01, *** *p* < 0.001, **** *p* < 0.0001, and “ns” indicates not significant.

The sublethal challenge was not lethal for all mice, with no significant weight loss observed. In lymph nodes, M2e5x VLP PLGA NPs were able to stimulate a higher expression of CD4^+^ T cells in lymph nodes and a higher expression of CD8^+^ cells in the lymph nodes and spleen (Figure 3A). M2e5x VLP + Alhydrogel^®^ + MPL-A^®^ PLGA NP-immunized mice showed significantly higher CD4^+^ and CD8^+^ T cells expression in both lymph nodes and spleens compared to M2e5x VLP. For the expression of intracellular cytokines IFN-γ and IL-4, M2e5x VLP PLGA NPs did not induce a higher expression of IFN-γ^+^ and IL-4^+^ T cells compared to M2e5x VLP (Figure 3B). In contrast, M2e5x VLP + Alhydrogel^®^ + MPL-A^®^ PLGA NP mice demonstrated a significantly higher expression of both IFN-γ^+^ and IL-4^+^ cells.

## 3. Discussion

M2e was selected as the vaccine antigen as it is highly conserved in influenza A. Further, a vaccine effectively priming an M2e-based response could lead to an efficacious, broadly protective pan-influenza A vaccine. M2e5x VLP was entrapped in polymeric PLGA nanoparticles to improve the immunogenicity and antigen-presentation of the M2e5x VLP. PLGA was selected as the polymer matrix for the vaccine nanoparticles as it is an FDA-approved biocompatible polymer. Past studies have shown that antigen presentation through the use of PLGA polymeric particles below 1 µm in size provides increased immune stimulation [41]. The potentially enhanced stimulation of dendritic cells could explain the increase in autophagy and nitric oxide production in DCs stimulated by M2e5x VLP PLGA NPs compared to the M2e5x VLP suspension. In general, nanoparticles of at least 500 nm are taken up by APCs [42]. Once the particles are internalized by APCs, the APCs travel to the lymphoid tissue, where the antigen can be processed and presented through the MHC Class II pathway [43]. During this process, some of the particles escape into the cytoplasm, releasing the antigen, which can then be presented through the MHC Class I pathway [43]. It has been previously shown that the uptake of PLGA particles approximately 300 nm to 1 µm in size by APCs can result in heightened MHC I and CD86 responses compared to particles above 1 µm in size [44]. Our M2e5x VLP PLGA nanoparticles and adjuvant nanoparticles, between 300 and 600 nm, fell within this size range and showed the potential for cross-presentation. 

Next, the sustained release profile of PLGA also likely contributed to enhanced antigen presentation. Previous studies with influenza peptide-loaded PLGA microspheres have demonstrated how the retention and slow hydrolysis of PLGA in the endosomes or cytoplasm of dendritic cells allow for the sustained release of peptide ligands for newly synthesized MHC class I molecules. Also, MHC class II presentation was also more sensitive and longer lasting when antigen delivery occurred via PLGA microspheres [45,46]. Similarly, our PLGA vaccine nanoparticles demonstrated a sustained release profile, suggesting that the heightened in vitro and in vivo immunogenicity of the M2e5x VLP PLGA nanoparticles compared to M2e5x VLP suspension may be linked to the sustained antigen release through slow degradation of the PLGA matrix. Next, the use of adjuvants, Alhydrogel^®^ and MPL-A^®^, were incorporated into the formulation to further enhance the immune response. Alhydrogel^®^ has been shown to induce a Th2-type response that effectively targets extracellular pathogens [47]. In contrast, the second adjuvant used, MPL-A^®^, is a TLR-4 ligand that stimulates CD4^+^ T cells to produce a Th1 response, which is advantageous for targeting intracellular pathogens [47]. Although influenza is an intracellular pathogen, inducing both Th1 and Th2 pathways is advantageous for inducing a balanced immune response against the virus [48]. Next, the advantage of fast-dissolving microneedles for vaccine delivery is that using biocompatible and water-soluble materials to produce needles that soften, dissolve, and deliver the payload within biological tissues upon penetration, prevent damages due to the mechanical forces associated with the application [49]. Therefore, the in vivo immunogenicity elicited by our microneedle vaccine groups demonstrates the potential of this tool for immunization through the skin. 

M2e-specific antibodies can protect against various IAVs through M2e immune-mediated protection [50]. Unlike HA-specific antibodies, M2e-specific antibodies are non-neutralizing but activate alternate pathways involving antibody-dependent cell-mediated cytotoxicity (ADCC). M2e IgG antibodies can induce Fc receptor-mediated phagocytosis by macrophages to aid in the clearance of the virus-infected cells [51]. For M2e5x VLP + Alhydrogel^®^ + MPL-A^®^ PLGA NP-immunized mice, there was a statistically significant increase in M2e-specific IgG1 compared to M2e5x VLP in week 4, which has previously been shown to elicit activation of alveolar macrophages to provide partial protection against lethal influenza infection in mice [52]. Additionally, M2e5x VLP+ Alhydrogel^®^ + MPL-A^®^ PLGA NP-immunized mice showed an increase in IgG2a, which peaked in week 4 and was still statistically significant in week 7, can help protect against influenza infection [52]. Furthermore, mice vaccinated with M2e5x VLP + Alhydrogel^®^ + MPL-A^®^ PLGA NPs showed significantly higher levels of lung IgA, an indicator of mucosal immunity [53,54], which is important for protection against viruses that target mucosal sites, such as in the nose and lungs. 

In research geared towards a universal influenza vaccine, there has been an increased focus on how CD4 and CD8 responses against conserved viral epitopes can confer heterosubtypic protection against influenza. Although antibodies are the prime standard for protection, the role of T cells against viruses such as influenza is significant. Studies that have immunized B cell-deficient mice have shown that primed M2e-specific CD4^+^ T cells can confer protection in the complete absence of protective antibodies through the activation of CD8 cells, regulation of innate immunity, and potential direct targeting of foreign pathogens [55]. The encapsulation of the antigen in a polymeric nanoparticle, as well as the incorporation of Alhydrogel^®^ and MPL-A^®^ likely contributed to an improved antigen presentation via MHC Class I and MHC Class II, resulting in a higher expression of both CD4^+^ and CD8^+^ T cells in lymph nodes and spleens. The higher expression of IFN-γ in splenocytes was significantly higher in the M2e5x VLP PLGA + Alhydrogel^®^ + MPL-A^®^ PLGA NP group, indicating a potentially heightened cell-mediated response, as confirmed through the elevation of CD8^+^ T cells. Levels of IL-4 were also statistically higher in the M2e5x VLP + Alhydrogel^®^ + MPL-A^®^ PLGA NP group, which promotes a Th2, or an antibody-driven response. Therefore, the production of both IFN-γ and IL-4-expressing T cells indicates a balanced Th1/Th2 response. Future studies will focus on assessing vaccine efficacy against lethal challenges with mouse-adapted influenza and assessing T and B cell subpopulations using flow cytometry. 

## 4. Materials and Methods

### 4.1. Formulation of M2e5x VLP-Loaded PLGA Nanoparticles

First, poly(lactic-co-glycolic acid) (PLGA; 70:25) was purchased from Evonik Industries (Essen, Germany). The PLGA powder (20 mg) was dissolved in 2.5 mL of dichloromethane (DCM; Fisher Scientific, Hampton, NH, USA) to form the O_1_ phase. Next, 0.5 mg of M2e5x VLP (kindly provided by Dr. Sang Moo Kang, Georgia State University) in 0.5 mL deionized water (W_1_) was added dropwise to the O_1_ phase. The production of M2e5x VLP has been described previously [25,56]. In brief, two human M2e (SLLTEVETPIRNEWGSRSN), one swine M2e (SLLTEVETPTRSEWESRSS), one type I avian M2e (SLLTEVETPTRNEWESRSS), and one type II avian M2e (SLLTEVETLTRNGWGCRCS), deemed M2e5x, and an oligomer-stabilizing domain GCN4 fusion protein, were linked to a cytoplasmic transmembrane domain of hemagglutinin. The construct was then expressed in Sf9 cells and purified through a sucrose gradient for 1 h. Lastly, the M2e5x VLP was adsorbed onto formvar carbon-coated copper grids for 15 min [25,56]. For formulating M2e5x VLP-loaded PLGA NPs, a PLGA:M2e5x VLP ratio of 40:1 was selected to increase encapsulation efficiency. The mixture was then emulsified using a probe homogenizer (Omni THQ Homogenizer, Omni International, Kennesaw, GA, USA) at 17,000 rpm for a total of 1 min to form the primary emulsion. The primary emulsion was then added dropwise to the second aqueous phase (W_2_), which consisted of 2% *w*/*v* polyvinyl alcohol (Sigma Aldrich, St. Louis, MO, USA) in 20 mL of deionized water. The mixture was then homogenized at 17,000 rpm for a total of 1 min to form the double emulsion. To facilitate particle size reduction, the W_1_/O_1_/W_2_ double emulsion was probe sonicated at 30% power for a total of 15 s (QSONICA, Newton, CT, USA). The previously mentioned procedure was also used to produce MPL-A^®^ (InvivoGen, San Diego, CA, USA) PLGA NPs. For Alhydrogel^®^ (InvivoGen) PLGA NPs, probe homogenization steps were carried out at 31,000 rpm. All formulations were put under magnetic stirring for 4–5 h to evaporate the DCM and harden the particles. Next, the nanoparticles were washed twice at 17,000 rpm for 12 min at 4 °C. The supernatant was then discarded, and the nanoparticles were resuspended in 2 mL of deionized water containing 2% *w*/*v* trehalose. The formulations were then lyophilized for 33 h and stored at 4 °C.

### 4.2. Particle Yield, Size, Charge, and Morphology

To calculate the total product yield of PLGA NPs, the following equation was used:

$$Product\;yield\;=\;\frac{Experimental\;product\;yield\;*\;100}{Theoretical\;product\;yield}$$

For determining particle size and charge (zeta potential), a Malvern Zetasizer Nano ZS (Malvern, UK) was used. The nanoparticles were visualized using scanning electron microscopy (SEM; Phenom benchtop SEM, Nanoscience Instruments, Phoenix, AZ, USA). 

### 4.3. Antigen Encapsulation Efficiency, Integrity, and Release

To determine encapsulation efficiency, 10 mg of M2e5x VLP PLGA nanoparticles were dissolved in 1 mL of DCM. The sample was then centrifuged at 4000 rpm for 10 min at RT. Next, the DCM was evaporated under a fume hood and the precipitated M2e5x VLP was resuspended in 1 mL of PBS and quantified using a Micro BCA™ Protein Assay Kit (Thermo Fisher, Waltham, MA, USA). The integrity of the encapsulated antigen in PLGA nanoparticles was assessed using a sodium dodecyl sulfate (SDS) PAGE. M2e5x VLP PLGA NPs were first dissolved in DCM. The DCM was evaporated, and the extracted M2e5x VLP was resuspended in 30 μL of PBS, followed by the addition of 10 μL of sample loading buffer. M2e5x VLP suspension was used as a control. All samples were run on an 8% pre-cast gel (Bio-Rad; Hercules, CA, USA). To determine M2e5x VLP release from the PLGA polymeric matrix, M2e5x VLP-loaded nanoparticles were dispersed in PBS (pH 7.4). Samples were taken at various time points over 6 days. 

### 4.4. Formulation and Characterization of Vaccine Nanoparticle-Loaded Microneedles

The formulation of PLGA nanoparticle-loaded dissolving MNs has been described previously [57]. In brief, trehalose (Sigma Aldrich) was dissolved in deionized water, after which doses equivalent to 20 μg M2e5x VLP, 50 μg alum, and 5 μg of MPL-A^®^ in PLGA NPs were suspended in the solution. Next, sodium hyaluronate (150 kDa; Lifecore Biomedical, Chaska, MN, USA) was then added to the suspension to form a 10% *w*/*v* gel. The formulation was mixed thoroughly, after which 25 mg of gel was added to a 10 × 10 microneedle array mold (Micropointe Technologies, Pte, Ltd., Singapore). The molds were then centrifuged at 4000 rpm for 20 min at 15 °C, and dried overnight in a desiccator. The next day, a concentrated HA solution was added to the molds to form a backing, which was then dried overnight. 

### 4.5. Induction of Autophagy

To evaluate the induction of autophagy in dendritic cells, DCs (DC2.4; Sigma Aldrich) were plated in 24-well plates at 50,000 cells/well. After reaching 70–75% confluency, the cells were pulsed with Fluzone (0.25 μg/well), M2e5x VLP (0.5 μg/well), Blank PLGA NPs (10 μg/well), and M2e5x VLP PLGA NPs (10 μg/well) overnight. The cells were washed 2× with PBS and stained according to protocol using a CYTO-ID^®^ Autophagy Detection Kit 2.0 (Enzo Life Sciences, Farmingdale, NY, USA). After 1 h, the cells were washed 2× twice with PBS, gently scraped, and analyzed using a flow cytometer (BD Accuri C6 flow cytometer).

### 4.6. Nitrite Production 

To assess the potential of M2e5x VLP PLGA NPs to induce the production of nitric oxide in immune cells, a Griess’ Assay was performed. First, dendritic cells were plated in a 24-well plate at a seeding density of 50,000 cells/well. After the cells reached approximately 70% confluency, various treatments were introduced to the cells: Cells Only, Lipopolysaccharide (LPS; Thermo Fisher) Blank PLGA NPs (10 μg/well), M2e5x VLP (0.5 μg/well), M2e5x VLP PLGA NPs (10 μg/well), and M2e5x VLP + Alhydrogel^®^ + MPL-A^®^ PLGA NPs (10 μg M2e5x VLP NPs, 50 μg Alhydrogel^®^ NPs, and 5 μg MPL-A^®^ NPs). This antigen:adjuvant ratio was selected as it would be the same ratio used for immunizations. The cells were incubated for 24 h after which the Griess’ Assay to assess nitric oxide production was performed. First, samples were centrifuged, and 50 μL of supernatant was transferred to a 96-well plate. Next, 50 μL of 1% sulphanilamide (Sigma Aldrich) was added to the wells and incubated at room temperature for 5 min in the dark. Next, 0.1% N-(1-naphthyl)ethylenediamine (NED; Sigma Aldrich) in 5% phosphoric acid (Sigma Aldrich) was added to the wells and placed on a shaker for 5 min. The plate was then read at 540 nm using a plate reader (Biotek Synergy, Agilent, Santa Clara, CA, USA). 

### 4.7. Surface Expression of Antigen-Presenting Molecules in Dendritic Cells

To assess antigen presentation in vitro, DCs were seeded in 24-well plates at a density of 50,000 cells/well. After reaching confluency, the cells were pulsed with various treatments: no treatment, Fluzone (0.25 μg/well), Blank PLGA NPs (10 μg/well), M2e5x VLP (0.5 μg/well), M2e5x VLP PLGA NPs (10 μg/well), and M2e5x VLP + Alhydrogel^®^ + MPL-A^®^ PLGA NPs (10 μg M2e5x VLP NPs, 50 μg Alhydrogel^®^ NPs, and 5 μg MPL-A^®^ NPs). The cells were incubated for 36 h. After the incubation, the cells were washed with PBS and gently scraped off the wells and resuspended in PBS and kept on ice. Next, anti-mouse MHC II and CD40 or MHC I and CD80 antibodies were added to the samples. Cells were incubated for 1 h and then washed 3× with PBS. The samples were analyzed using flow cytometry. 

### 4.8. Immunization, Blood Collection, and Challenge

For animal studies, 4–6-week-old female Swiss Webster (CFW) mice (Charles River, Wilmington, MA, USA) were immunized as follows (*n* = 5): Naïve (no treatment), Fluzone (1.5 μg), Blank PLGA NPs (900 μg unloaded NPs), M2e5x VLP (20 μg), M2e5x VLP PLGA NPs (20 μg M2e5x VLP), and M2e5x VLP + Alhydrogel^®^ + MPL-A^®^ PLGA NPs (20 μg M2e5x VLP, 50 μg alum, and 5 μg of MPL-A^®^). All groups, except Naïve and Fluzone, received treatment via the dissolving microneedles. Fluzone mice were immunized intraperitoneally. Mice were immunized using a prime-boost strategy in weeks 0 and 3. Blood was collected intermittently in weeks 1, 4, and 7. Whole blood was centrifuged at 1000× *g* to isolate the serum, which was then collected and stored at −80 °C until use. In week 8, mice were challenged intranasally with 50 μL of 0.5 × LD_50_ of A/Philippines/2/82 (H3N2). A sublethal challenge was used to assess M2e priming in immunized groups as well as having a surviving naïve control for T cell analysis. The mice were monitored for 14 days. In week 10, the mice were sacrificed. Organs including lymph nodes, spleens, and lungs were then harvested. 

### 4.9. Enzyme-Linked Immunosorbent Assay (ELISA) to Evaluate M2e-Specific Antibody Responses

M2e-specific serum antibody responses were evaluated using enzyme-linked immunosorbent assay (ELISA). First, M2e was coated on high-binding 96-well plates at a concentration of 0.2 μg/well. For the positive control, Fluzone, wells were coated with 0.2 μg/well of Fluzone. The plates were incubated at 4 °C overnight. The next day, plates were washed 3× with PBST (PBS + 0.1% *v*/*v* Tween^®^ 20; Sigma Aldrich) and then blocked with 150 μL of 3% bovine serum albumin (BSA; Sigma Aldrich) in PBS for 2.5 h at RT. Next, plates were washed 3× with PBST, and diluted serum samples (50 μL/well) starting at a 1:10 dilution were added to plates and incubated at 37 °C for 1.5 h. The plates were then washed and horseradish peroxidase (HRP)-conjugated goat anti-mouse IgG, IgG1, and IgG2a antibodies (Thermo Fisher) were added to the wells incubated at 37 °C for 1.5 h. The plates were then washed prior to the addition of 50 μL/well of 3,3′,5,5′-tetramethylbenzidine (TMB; Thermo Fisher). The plates were then incubated at room temperature for 7 min followed by the addition of 50 μL/well of 0.3 M sulfuric acid (H_2_SO_4_). The absorbance was read at 450 nm. For assessing IgA in lungs, after sacrifice, lung tissue from mice was removed and homogenized. Tissue was then centrifuged at 1200 rpm, and supernatants were collected and frozen at −80 °C. For analysis, supernatants from lung homogenates were ten-fold serially diluted, starting at a 1:10 dilution. The same method for ELISA for serum samples was utilized for lung supernatants, with a 1:3000 dilution of HRP-conjugated IgA. 

### 4.10. Evaluation of T Cell Responses in Immune Organs

After animals were sacrificed, immune organs such as spleens and lymph nodes were harvested. In brief, the spleens were passed through 40-micron cell strainers (Fisher Scientific) to form a single-cell suspension, and centrifuged at 1200 rpm for 10 min. Next, 1 mL of ACK lysis buffer was added to the splenocytes and incubated for 3 min. Next, the cells were centrifuged. Finally, the cell pellets were resuspended in DMEM containing 70% fetal bovine serum (FBS; Thermo Fisher) and stored at −80 °C until analysis. Similarly, lymph nodes were passed through a 40-μm cell strainer, centrifuged, and resuspended in DMEM containing 70% FBS before being frozen at −80 °C. To assess cell surface markers CD4 and CD8 (Thermo Fisher), and intracellular cytokines interferon-gamma (IFN-γ) and interleukin-4 (IL-4), cell suspensions were thawed and centrifuged at 1200 rpm for 10 min. Lymph node and spleens cells from M2e5x VLP antigen-immunized groups were pre-stimulated with 1 μg of M2e protein (provided by Dr. Sang-Moo Kang) in 24-well plates. Immune organs from mice immunized with Fluzone were pre-stimulated with 0.5 μg quadrivalent Fluzone. Next, cells were supplemented with 50 μM β-mercaptoethanol (Thermo Fisher) and incubated overnight at 37 °C. The following day, 100 μL of 1× protein inhibitor Brefeldin A (Thermo Fisher) was added to each well. The plates were incubated at 37 °C for 1 h after which the cells were stained with the CD4 and CD8 for 1 h. For intracellular markers, cells were fixed and permeabilized using the Fixation and Permeabilization kit (Thermo Fisher) following the manufacturer’s instructions. Next, cells were stained with fluorescently labeled anti-mouse IFN-γ and IL-4. The samples were then incubated on ice for 1 h. The cells were washed and analyzed using flow cytometry. 

### 4.11. Statistical Analysis

Data analyses were performed on GraphPad Prism Version 9.2. and expressed as mean values with the standard deviation (SD). For multiple comparisons, one-way analysis of variance (ANOVA) was performed with Tukey’s test for post-hoc analysis. A *p*-value < 0.05 was considered to be statistically significant.

## 5. Conclusions

The M2e5x VLP formulated into PLGA nanoparticles and combined with Alhydrogel^®^ + MPL-A^®^ nanoparticles was shown to be an immunogenic M2e-based influenza vaccine. The nanoparticles induced significant autophagy and nitrite production, and antigen presentation in dendritic cells. Furthermore, the M2e5x VLP PLGA NPs along with adjuvants demonstrated enhanced M2e-specific IgG, IgG1, IgG2a, and IgA responses compared to the M2e5x VLP suspension. Additionally, the vaccine nanoparticles with adjuvants elicited a significant expression of CD4^+^ and CD8^+^ T cells in immunized mice. Hence, the incorporation of M2e5x VLP into a biodegradable PLGA polymeric matrix with adjuvants and delivered non-invasively through the transdermal route using a fast-dissolving microneedle shows the potential for an innovative pan-influenza A vaccine capable of inducing robust immunity against the influenza virus.

## Data Availability

The data presented in this study is available upon request from the corresponding author.
